# Cleaning Teeth and Dentifrices

**Published:** 1867-12

**Authors:** G. A. Mills


### CLEANING TEETH AND DENTIFRICES.

BY G. A. MILLS.

Read before the Brooklyn Dental Association, May 1st, 1867.

Is there a subject more closely allied to the Dentist than that of cleanliness? I commenced this paper under the conviction that it was a blessed thing to be clean, to say nothing of its health-giving properties. If any desire to feel that they are clean, I would advise them to take the benefit of a Turkish bath, as I did an hour previous to the writing of this paper, and think, if I succeed in anything that will be suggestive, it will be due in a great measure to cleanliness. Who, more than the Dentist, needs to be clean in the body, yea, in spirit? then all the works of our hands *will* be clean also, and not till then. We have heard much from our worthy brother Atkinson about clean operations, and receiving clean money. There is a world of truth in this idea. The oft-repeated saying, "that cleanliness is next to godliness," should be the talisman of our daily life.

I am quite sure, the matter of cleaning teeth is not so fully comprehended by us as it deserves to be, so far as my own experience and observation extends. I well recollect my first experience in cleaning teeth literally. It was while studying with my first tutor in Dentistry. I took them to the laboratory (I speak of extracted teeth) and with stick and pumice for a time, then with the lathes and whiting and brushes, I succeeded in producing a beautifully polished tooth. I was surprised to find the human teeth so susceptible to a fine polish. I have since that time resolved, and re-resolved, to produce the same results in the mouth; but I am sorry to say it is a duty long neglected. Dare I say, or any person, it cannot be done? on the contrary, it can; and this I have proved since our last meeting, much to my joy, and that of the patient. Why has the importance of thus polishing the teeth, been so seldom brought to our notice. Should this meet the eyes of the Dental profession, I have no doubt there are legions who would say, *this is my daily practice.* How many Dentists do you think there are, who clean teeth in the strict sense of the term? Not more than one in a thousand. This may seem a very extravagant statement, but I am willing it should go out. This operation is less perfectly performed than any we are called upon to do. I am strongly inclined to believe that a large per cent. of the teeth are lost to the possessor for the want of proper cleaning, both by neglect on *their part*, and that of the *Dentist.* This, sir, should not be placed at our door; but, *in part*, it belongs there, and justly too. Why? Simply because we deceive the patients by educating them falsely. When *we* urge upon them the necessity of keeping their teeth clean, we should first give them an example to follow, by first putting them in proper order. How do we clean teeth? By removing all the foreign matter we see and leaving that we do not see, because we do not look where it is. There is much iniquity hidden from the natural eye, but it is a growing worm and the yellow leaf is too soon seen. A large proportion of the destroyer lies hidden under the margin of the gums, sometimes producing irritation to such an extent as to cause the death of the ligamentum dentium; then follows the death of the alveolar process; then loosened teeth, which soon become useless. Notwithstanding, *some* of the would-be knowing ones, scout the idea of magnifying glasses in dental operations. It is my opinion that we would be better Dentists if we would use them more, thereby enabling us to be more perfect in the operation of cleaning teeth. I might well say something upon the cleaning of cavities preparatory to filling, but will not dwell upon it at this time. I have asked how we should clean teeth? Now I will answer, how in my opinion, it should be done. I would first remove all the crusty deposit with as convenient an instrument as possible, taking pains to remove nothing but a DEPOSIT. I would then work upon a SINGLE tooth with a soft stick or cork, together with polishing stones, pumice, calcined brickstone, cotton and chamois skin, etc., until I had succeeded in producing a brilliant polish, which can always be done. Take one tooth and work upon it until you accomplish the desired result. Bring that *one* up to your highest ideal, then examine it under the glass and compare it with the adjoining one,—and if *you* are clean,—the rest may be made so. Some will ask, pray how long a time would one spend? Now if I should answer this question according to the ideas of nine-tenths of the human race, I would say just as long as it would *pay*. Would to God that we might accept the belief that ALL good works never go unrewarded. When we come to this we shall follow in the strict line of duty, and cease to worry about the so-called *almighty dollar*. "The seed of our Heavenly Father never go begging bread." Since the last meeting of this society I have taken into my hands two cases, and am determined to see what results I can produce. As far as I have gone the results secured are exceedingly satisfactory to the patients and myself. In one case the patient is more than usually particular about trying to keep the teeth clean. I first examined them by the natural vision, and could see what seemed to be a slight stain at the margin of the gums. I then took my magnifier, and was surprised to see the change; what seemed to be a slight stain, proved to be a calcareous deposit of no small amount. I allowed the patient to look for herself, and she was soon convinced that there was work to be done. I then took an excavator and examined it, and proved by the delicate sense of touch that *a deposit was there.* The labial surfaces and all others the brush could reach, were beautifully polished. This patient insisted that I should spare no pains or expense to put them in thorough order. A great deal of time will be required to produce good results, but we should never let this deter us from doing our whole duty. There are often indentations, that should be removed, because if left they will be the first spots to receive new deposits, and all will agree that smooth surfaces are much easier kept clean than rough ones. Simply removing the deposit is not enough to secure the desired end, for there will be more or less minute portions of foreign matter adhering to the teeth, making a future accumulation more certain; with a perfectly smooth surface the process of mastication will do much to keep them cleanly. Teeth once brought to this polished surface, will be in a far better condition to commit to the patient's care, and the means employed to do this, will be instructing them in the right direction.

Now will come the oft-repeated inquiry, what shall I use besides the brush? A brush will do much to remove deposits, but there are many reasons why a proper Dentifrice should be used in connection, which are apparent to you all, and should be impressed on the patient's mind.

To go back into the past and rehearse the multiplied list of Dentifrices that have been presented up to the present time, would prove itself too great a task for my time and your patience. Almost a numberless variety have been brought to the notice of the public, and could it have been that half the recommendations accompanying them were true, our mission would have ceased to be a *mission* any longer. I think we cannot be too severe in crying down the many nostrums advertised upon the curbstones and fences of our cities. If we know what is a suitable preparation let us recommend it, and encourage any respectable member of our profession who is willing to make a specialty of the manufacture of a proper Dentifrice, that we can heartily and conscientiously endorse; with two or three exceptions I have never met with a Dentifrice *circulated* through the country for sale, that had the signature of a responsible Dentist. And doubtless, for this reason, the statements accompanying the preparations were of such a nature that no *decently truthful* Dentist could endorse them. We are all well aware that there is no magic in a proper Dentifrice with which we supply our patients. The simpler the preparation, the better it is for the masses. It is well known that orris root, prepared chalk and castile soap, is a simple and as useful a formulæ when properly prepared, as any we can recommend. I do not believe in putting into the hands of patients without discrimination, cuttle fish or pumice, even mixed with the formulæ just given. But still worse than this, there are some (I trust but few,) who put pumice alone into their patient's hands (*to be used with a brush like a simple Dentifrice*), at their pleasure. Such Dentists, I think, had better go back to first principles and learn their lessons better. Another Dentifrice called charcoal paste, I do not think a proper article to be generally used. I would raise the same objections as with pumice, it is too sharp for the teeth for constant use. I believe it is the general opinion of the thinking portion of our profession that medicated Dentifrices for general use are not needed. The American Dental Association at Boston, last year, denounced in toto the use of mouth washes in any form as toilet articles.

In closing I would refer to the Tooth Tablets originated by our worthy brother, Dr. I. W. Lyon, and I take great pleasure in giving them my most hearty recommendation, as being (taken as a whole) superior to any thing I have met as a Dentifrice. They contain no magic, but are a simple, safe, neat and very convenient preparation ; giving as far as my patients are concerned, universal satisfaction. For my own part, I see no reason why we cannot consistently give them our hearty approval, and as Dr. Lyon is to make this his specialty from to-day, I believe in bidding him God speed in his efforts to clean the teeth of the whole human race.

